# Aviculin Isolated from *Lespedeza cuneata* Induce Apoptosis in Breast Cancer Cells through Mitochondria-Mediated Caspase Activation Pathway

**DOI:** 10.3390/molecules25071708

**Published:** 2020-04-08

**Authors:** Dahae Lee, Yong Hoon Lee, Kwang Ho Lee, Bum Soo Lee, Akida Alishir, Yoon-Joo Ko, Ki Sung Kang, Ki Hyun Kim

**Affiliations:** 1 College of Korean Medicine, Gachon University, Seongnam 13120, Korea; pjsldh@naver.com; 2 School of Pharmacy, Sungkyunkwan University, Suwon 16419, Korea; yhl2090@naver.com (Y.H.L.); sholaly@naver.com (K.H.L.); kosboybs@naver.com (B.S.L.); akida.alishir@gmail.com (A.A.); 3 Laboratory of Nuclear Magnetic Resonance, National Center for Inter-University Research Facilities (NCIRF), Seoul National University, Gwanak-gu, Seoul 08826, Korea; yjko@snu.ac.kr

**Keywords:** *Lespedeza cuneata*, aviculin, MCF-7 human breast cancer cells, apoptosis, caspase

## Abstract

The global incidence of breast cancer has increased. However, there are many impediments to the development of safe and effective anticancer drugs. The aim of the present study was to evaluate the effect of aviculin isolated from *Lespedeza cuneata* (Dum. Cours.) G. Don. (Fabaceae) on MCF-7 human breast cancer cells and determine the underlying mechanism. Using the bioassay-guided isolation by water soluble tetrazolium salt (WST-1)-based Ez-Cytox assay, nine compounds (four lignan glycosides (**1**–**4**), three flavonoid glycosides (**5**–**7**), and two phenolic compounds (**8** and **9**)) were isolated from the ethyl acetate (EA) fraction of the *L. cuneata* methanolic extract. Of these, aviculin (**2**), a lignan glycoside, was the only compound that reduced metabolic activity on MCF-7 cells below 50% (IC_50_: 75.47 ± 2.23 μM). The underlying mechanism was analyzed using the annexin V Alexa Fluor 488 binding assay and Western blotting. Aviculin (**2**) was found to induce apoptotic cell death through the intrinsic apoptosis pathway, as indicated by the increased expression of initiator caspase-9, executioner caspase-7, and poly (ADP-ribose) polymerase (PARP). Aviculin (**2**)-induced apoptotic cell death was accompanied by an increase in the Bax/Bcl-2 ratio. These findings demonstrated that aviculin (**2**) could induce breast cancer cell apoptosis through the intrinsic apoptosis pathway, and it can therefore be considered an excellent candidate for herbal treatment of breast cancer.

## 1. Introduction

The incidence of breast cancer is increasing rapidly [1]. Of all cancers in women, breast cancer has the highest incidence rate and highest mortality rate [2]. Cancer remains a cause for concern despite the increase in physical and chemical therapies, and there are still many barriers to the treatment of breast cancer, such as adverse effects, arising from radiotherapy, surgery, and chemotherapy/hormonotherapy [3]. Therefore, pharmaceutical industries show growing interest in different options to treat cancer. One example of those options can be considered as herbal treatment or traditional medicine. If the given natural source represents significant anticancer activity, it can be used as a prophylaxis or adjuvant for cancer treatment.

Previous pharmacological studies showed that extracts of *Lespedeza cuneata* can be used to treat hematuria and insomnia. In addition, extracts of *L. cuneata* were reported to have hepatoprotective and antidiabetic effects [4], anticancer and antioxidative activity, and inhibitory effects on cell proliferation [5]. The useful cosmetic effects of *L. cuneata* extracts, such as antiwrinkle, antimelanogenic, anti-aging, and anti-oxidative effects, have also been reported [6,7]. *L. cuneata* also showed pharmacological effects against male and female diseases, such as protective effects against testicular and ovarian diseases [8,9]. A phytochemical analysis of *L. cuneata* revealed the presence of various types of compounds, such as lignans, phenylpropanoids, pinitols, flavonoids, and tannins [10]. Flavonoids, one of the major classes of compounds present in *L. cuneata*, were reported to have inhibitory effects on NO production, which causes inflammation [11]. In addition, lignans, also one of the major classes of compounds in *L. cuneata*, have hepatoprotective effects [12].

As part of the continuing research into bioactive compounds from Korean medicinal plant sources [13,14,15,16,17], the MeOH extract of the aerial portion of *L. cuneata* was found to exhibit cytotoxic effects on human ovarian carcinoma cells [5]. In our recent study, bioassay-guided fractionation of the MeOH extract using repeated chromatography resulted in the isolation of (−)-9′-*O*-(α-l-rhamnopyranosyl)lyoniresinol, which inhibited the proliferation of A2780 human ovarian carcinoma cells through the induction of apoptosis [5]. In our ongoing study of *L. cuneata*, the phytochemical analysis of the *L. cuneata* MeOH extract led to the isolation of two new lignan glycosides along with three known lignan glycosides and nine known flavonoid glycosides, some of which exhibited weak cytotoxicity against four human breast cancer cell lines (Bt549, MCF-7, MDA-MB-231, and HCC70) [18]. In the current study, we found in a screening test that the MeOH extract of *L. cuneata* exerted inhibitory effect on metabolic activity in MCF-7 human breast cancer cells, which led us to perform further phytochemical analysis and identify the bioactive compound(s) responsible for these effects. Bioassay-guided fractionation and chemical investigation of the MeOH extract led to the isolation of nine compounds (four lignan glycosides (**1**–**4**), three flavonoid glycosides (**5**–**7**), and two phenolic compounds (**8** and **9**)). The structures of the isolated compounds were identified by LC-MS analysis and their spectroscopic data and physical data were compared with previously reported values. In this paper, we report the isolation and structural characterization of compounds **1**–**9** and their effect on metabolic activity with respect to their apoptosis effects in MCF-7 breast cancer cell line.

## 2. Results

### 2.1. Effects of Four L. cuneata MeOH Extract Fractions (n-Hexane, CH_2_Cl_2_, EtOAc, and n-BuOH Fractions) on Metabolic Activity in MCF-7 Human Breast Cancer Cells

**Figure 1:**
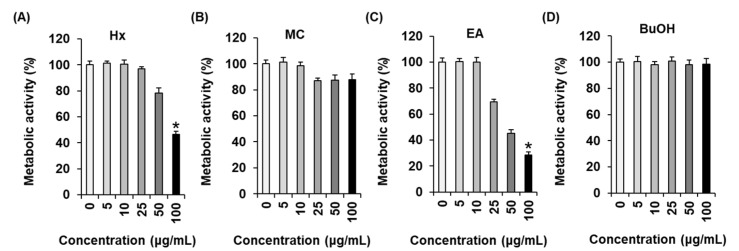
Effects of *L. cuneata* methanolic extract fractions on metabolic activity in MCF-7 human breast cancer cells. The *n*-hexane (HX) (**A**); dichloromethane (MC) (**B**); ethyl acetate (EA) (**C**); and *n*-butanol (BuOH) (**D**) fractions exerted inhibitory effect on metabolic activity in MCF-7 cells. Control cells were treated with 0.5% DMSO. The experiment was performed in triplicate, and the error bars represent the standard deviation of samples (*n* = 3). * *p* < 0.05 compared with the control.

The MeOH extract of *L. cuneata*, which has inhibitory effect on metabolic activity in MCF-7 cells, was sequentially solvent-partitioned with *n*-hexane, CH_2_Cl_2_, EtOAc, and *n*-BuOH to obtain the *n*-hexane (HX), dichloromethane (MC), ethyl acetate (EA), and *n*-butanol (BuOH) fractions, respectively. The water-soluble tetrazolium salt (WST-1)-based Ez-Cytox assay was performed for the four fractions to evaluate their effects on metabolic activity in MCF-7 cells. The HX fraction moderately reduced metabolic activity in MCF-7 cells (Figure 1A, IC_50_: 94.37 ± 3.22 μg/mL), and the MC fraction did not affect metabolic activity in MCF-7 cells (Figure 1B). The EA fraction potently suppressed metabolic activity in MCF-7 cells in a concentration-dependent manner (Figure 1C, IC_50_: 45.03 ± 2.24 μg/mL), however, the BuOH fraction did not affect metabolic activity in MCF-7 cells (Figure 1D). Based on these results, we investigated the active compounds in the most active fraction, the EA fraction, which contributed to the inhibition of metabolic activity in MCF-7 cells.

### 2.2. Isolation and Identification of Compounds from the EtOAc Fraction

**Figure 2:**
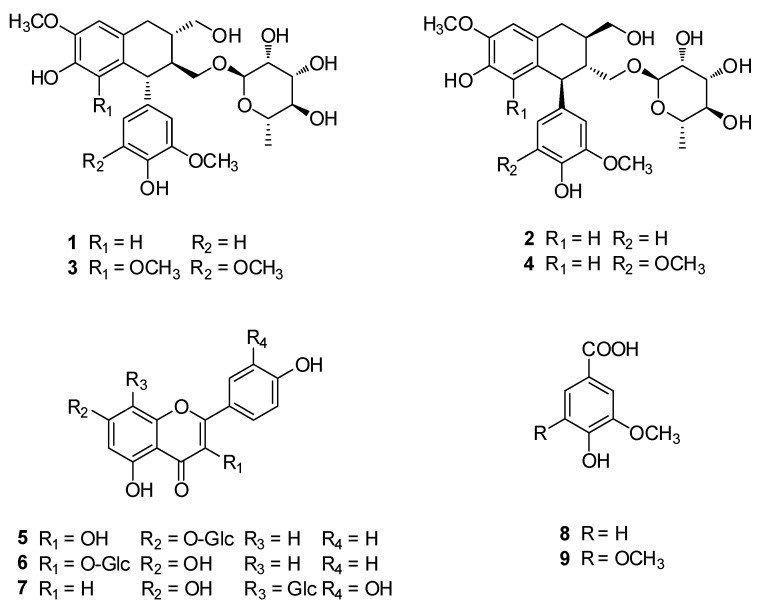
Chemical structures of compounds **1**–**9**.

Chemical investigation of the EA fraction using repeated column chromatography and HPLC purification led to the isolation of nine compounds: four lignanosides (**1**–**4**), three flavonoid glycosides (**5**–**7**), and two phenolic compounds (**8** and **9**). As shown in Figure 2, the isolated compounds were identified as (−)-(8*S*,7’*R*,8’*S*)-isolariciresinol-9’-*O*-α-l-rhamnoside (**1**) [19], aviculin (**2**) [19], (−)-9′-*O*-(α-l-rhamnopyranosyl)lyoniresinol (**3**) [20], (+)-5′-methoxyisolariciresinol-9′-*O*-α-l-rhamnoside (**4**) [19], kaempferol-7-*O*-β-d-glucopyranoside (**5**) [21], astragaline (**6**) [22], orientin (**7**) [23], vanillic acid (**8**) [24], and syringic acid (**9**) [25] by LC/MS analysis and the comparison of their spectroscopic data, including the ^1^H and ^13^C-NMR spectra (Figures S1–S10) , and physical data with previously reported values.

### 2.3. Effects of Compounds ***1**–**9*** on Metabolic Activity in MCF-7 Human Breast Cancer Cells

**Figure 3:**
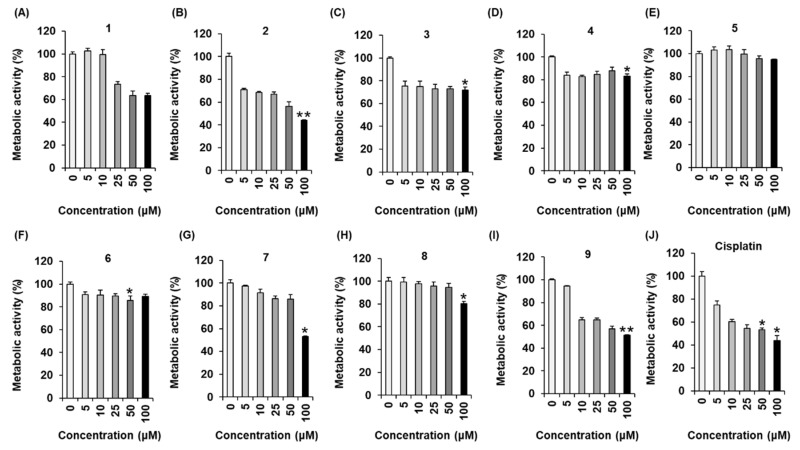
Effects of the isolated compounds **1**–**9** (**A**–**I**) and cisplatin (**J**) on metabolic activity in MCF-7 human breast cancer cells. Control cells were treated with 0.5% DMSO. The experiment was performed in triplicate, and the error bars represent the standard deviation of samples (*n* = 3). * *p* < 0.05, ** *p* < 0.01 compared with the control.

All the isolates (**1**–**9**) were evaluated for their effect on metabolic activity in MCF-7 human breast cancer cell lines. The percentages of metabolic activity decreased to 63.59% ± 2.05% after treatment with 100 μM (−)-(8*S*,7’*R*,8’*S*)-isolariciresinol-9’-*O*-α-l-rhamnoside (**1**) (Figure 3A), whereas aviculin (**2**) potently suppressed metabolic activity in MCF-7 cells in a concentration-dependent manner (Figure 3B, IC_50_: 75.47 ± 2.23 μM). Percentile values of metabolic activity were declined to 71.67% ± 2.85% after treatment with 100 μM (−)-9′-*O*-(α-l-rhamnopyranosyl)lyoniresinol (**3**) (Figure 3C). Metabolic activity was reduced to 83.08% ± 1.82% after treatment with 100 μM (+)-5′-methoxyisolariciresinol-9′-*O*-α-l-rhamnoside (**4**) (Figure 3D). Likewise, kaempferol-7-*O*-β-d-glucopyranoside (**5**) and astragaline (**6**) showed metabolic activity decreased to 94.96% ± 0.43% and 89.05% ± 2.03%, respectively, after treatment with 100 μM (Figure 3E,F). Orientin (**7**) showed suppressed metabolic activity to 52.94% ± 0.62% after treatment with 100 μM. (Figure 3G). The percentages of metabolic activity decreased to 80.29% ± 1.85% after treatment with 100 μM vanillic acid (**8**) (Figure 3H). Finally, syringic acid (**9**) showed reduced metabolic activity to 51.27% ± 0.47% after treatment with 100 μM (Figure 3I). It is concluded that aviculin (**2**) has an inhibitory effect on metabolic activity in MCF-7 cells, but it is not as effective as the conventional chemotherapeutic agent cisplatin, which was the positive control (Figure 3J, IC_50_: 67.26 ± 3.01 μM). In our SAR analysis, the comparison of the relative activity levels of compounds **1**/**2** vs. **3**/**4** and **8** vs. **9** demonstrated that the methoxy groups at aromatic rings can influence the potency. In particular, the difference in the relative effect of compounds **1** vs. **2** on metabolic activity in MCF-7 cells suggested that the stereochemistry of 8*R*,7’*S*,8’*R* in the lignan-type compounds **1**–**4** plays a role in potency. We then determined whether the inhibitory effect of aviculin (**2**) on MCF-7 cells was associated with apoptosis.

### 2.4. Effects of Aviculin (***2***) on Nuclear Morphologies of MCF-7 Human Breast Cancer Cells

**Figure 4:**
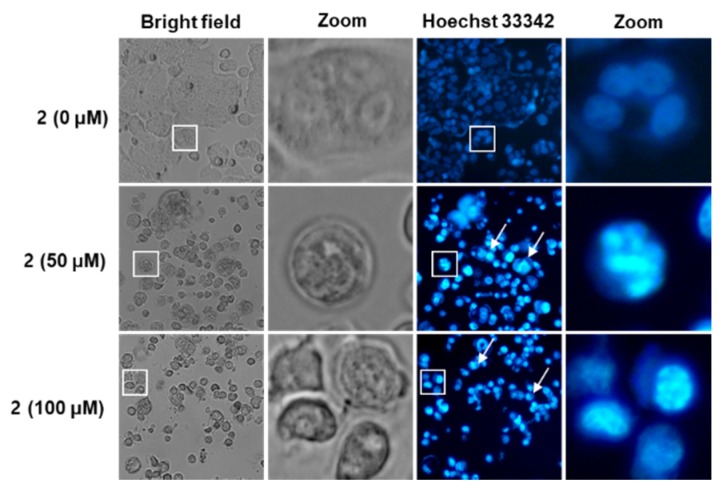
Effects of aviculin (**2**) on nuclear morphologies of MCF-7 human breast cancer cells. Representative fluorescence microscopy images of Hoechst 33342 stained cells (20× magnification). Control cells were treated with 0.5% DMSO. Several independent images were photomicrographed and representative images for each condition are shown. Condensed and bright nuclei (white arrow) in the enlarged image marked in white square are indicative of apoptotic cell death.

Control MCF-7 cells had a normal morphology, while MCF-7 cells incubated with aviculin (**2**) showed marked cellular blebbing and shrinkage (Figure 4). To observe nuclear condensation of MCF-7 cells incubated with aviculin (**2**), cell staining with Hoechst 33342, a blue fluorescent cell permeable dye that binds nucleic acid, was performed. As shown in Figure 4, nuclei were lightly stained blue in control MCF-7 cells, while MCF-7 cells incubated with aviculin (**2**) deeply stained blue due to staining of the condensed nuclei of apoptotic cells. These changes of nuclear morphologies of MCF-7 cells suggest that aviculin (**2**) induces apoptotic cell death.

### 2.5. Effects of Aviculin (***2***) on Apoptotic Cell Death in MCF-7 Human Breast Cancer Cells

**Figure 5:**
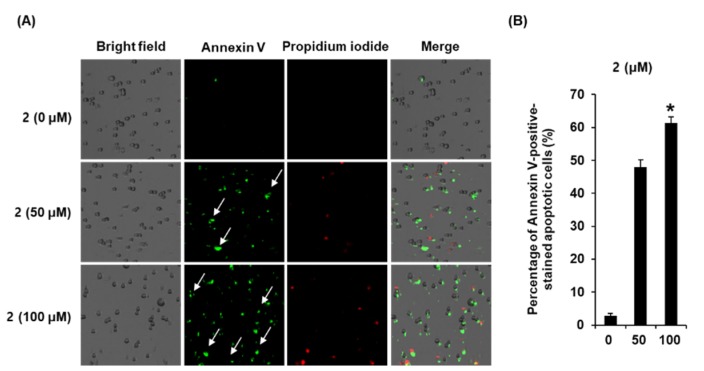
Effects of aviculin (**2**) on apoptotic cell death in MCF-7 human breast cancer cells. (**A**) Representative images of apoptotic cells stained with annexin V (green) and propidium iodide (red). (**B**) Percentage of annexin V-positive apoptotic cells. Control cells were treated with 0.5% DMSO. The same number of cells for the control and sample were stained and analyzed. Ten different fields (40× magnification) per slide were taken with Tali Image-Based Cytometer and representative fields for each condition are shown. The experiment was performed in triplicate, and the error bars represent the standard deviation of samples (*n* = 3). * *p* < 0.05 compared with the control.

Staining with annexin V and propidium iodide was performed to evaluate the apoptosis-inducing effects of 50 and 100 μM aviculin (**2**) in MCF-7 cells. The apoptotic cell death of MCF-7 cells caused by aviculin (**2**) is shown in Figure 5. The percentages of annexin V-positive cells increased to 46.23% ± 4.32% and 59.33% ± 2.51% after treatment with 50 and 100 μM aviculin (**2**), respectively. These results are consistent with the results of cell staining with Hoechst 33342.

### 2.6. Effects of Aviculin (***2***) on the Expression of Apoptosis-related Proteins in MCF-7 Human Breast Cancer Cells

**Figure 6:**
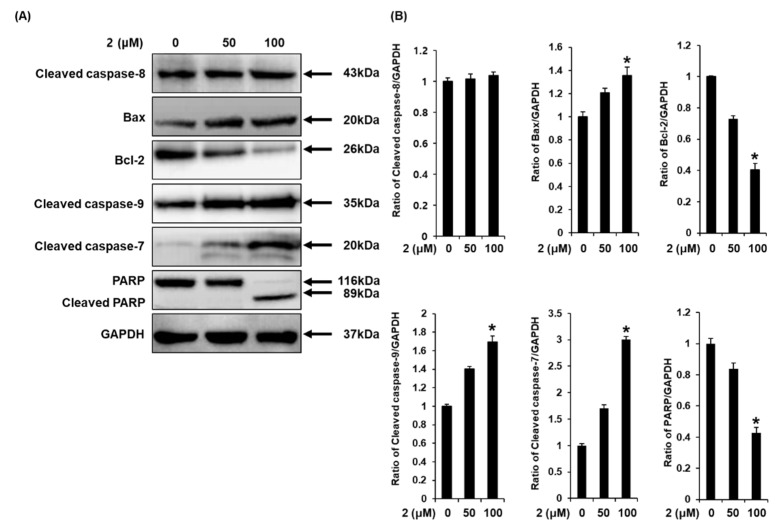
Effects of aviculin (**2**) on the expression of apoptosis-related proteins in MCF-7 human breast cancer cells. (**A**) Representative images of Western blotting for cleaved caspase-8, Bax, Bcl-2, cleaved caspase-9, cleaved caspase-7, PARP, and GAPDH in MCF-7 cells treated with aviculin (**2**) (50 and 100 μM) for 24 h. GAPDH was used as the internal control. (**B**) Each bar graph presents the densitometric quantification of the Western blotting bands. Control cells were treated with 0.5% DMSO. The experiment was performed in triplicate, and the error bars represent the standard deviation of samples (*n* = 3). * *p* < 0.05 compared with the control.

The mechanisms underlying the effects of aviculin (**2**) on apoptotic cell death in MCF-7 human breast cancer cells were examined through Western blotting. In an analysis of the levels of apoptotic initiator caspases, treatment with aviculin (**2**) upregulated cleaved caspase-9 in a concentration dependent manner, whereas it did not affect the level of cleaved caspase-8 (Figure 6). Moreover, treatment with aviculin (**2**) increased cleaved caspase-7 (an apoptotic effector caspase) in a concentration dependent manner. In the expression change of full-length poly (ADP-ribose) polymerase (PARP), a specific substrate of caspase-7, it was only cleaved in fragments of 89 kDa at treatment with 100 μM aviculin (**2**). Treatment with aviculin (**2**) also increased the expression of the pro-apoptotic protein Bax, but decreased the expression of the anti-apoptotic protein Bcl-2 in a concentration dependent manner. These results suggest that aviculin (**2**) induces apoptosis through the intrinsic, mitochondria-mediated pathway.

## 3. Discussion

In our previous study, (−)-9′-*O*-(α-l-rhamnopyranosyl)lyoniresinol (**3**), a lignan with rhamnose, induced apoptosis in A2780 human ovarian carcinoma cells through the induction of the extrinsic apoptotic signaling pathway via the activation of caspase-8. In our continuing research into the anticancer activity of compounds isolated from *L. cuneata*, the EA fraction from the *L. cuneata* MeOH extract was found to exhibit inhibitory effect on metabolic activity in MCF-7 human breast cancer cells. These results reveal that the EA fraction of the *L. cuneata* MeOH extract is not only toxic to human ovarian carcinoma cells, but also to human breast cancer cells. The subsequent chemical investigation of the EA fraction using repeated column chromatography and HPLC purification led to the isolation of four lignanosides (**1**–**4**), three flavonoid glycosides (**5**–**7**), and two phenolic compounds (**8** and **9**). In the in vitro studies using MCF-7 cells, aviculin (**2**), a lignan glycoside, was the only compound that reduced metabolic activity in MCF-7 cells by more than 50% (IC_50_: 75.47 ± 2.23 μM). Despite the different cell types used in this study and our previous study, the inhibitory effect of lignan compounds on metabolic activity in MCF-7cells was confirmed, supporting our previous study. 

In the development of better and safer anticancer drugs, lignan compounds isolated from various plants have been studied as candidate compounds owing to their anticancer activity [26,27]. In addition, lignan compounds have been used as starting compounds for the semi-synthesis of anticancer drugs [26,28]. A previous study reported that aviculin isolated from *Scurrula atropurpurea* (Loranthaceae) inhibited cancer cell invasion in MM1 rat mesothelial cells [29]. To the best of our knowledge, there are no papers that report the inhibitory effect of aviculin on metabolic activity in cancer cells. 

Our study is the first to report that aviculin (**2**) suppresses metabolic activity in breast cancer cells through the induction of apoptosis. To further investigate the mechanism of cell death induced by aviculin (**2**), annexin V, which is known to bind to phosphatidylserine and identify apoptotic cells, was used. Apoptosis, or genetically programmed cell death, is known to have a crucial role in the development of cancer [30,31]. Western blotting was used to determine whether apoptosis was induced via the extrinsic or intrinsic apoptosis pathways. Aviculin (**2**) did not induce the extrinsic apoptosis pathway. The extrinsic pathway can be triggered by binding of ligands to death receptors and caspase-8 activation [32]. In the present study, aviculin (**2**) did not change the expression of cleaved caspase-8, whereas it induced apoptosis via the intrinsic apoptosis pathway through the activation of caspase-9, resulting in changes in the levels of cleaved caspase-7 and cleaved PARP. However, 89 kDa fragment of PARP was only detected at treatment with 100 μM aviculin (**2**). During apoptosis, PARP is an essential hallmark that is completely cleaved when treated with concentrations that induce high rate of cell death [33]. The lack of cleavage of PARP after treatment with 50 μM aviculin (**2**) is considered as incomplete apoptosis or other types of cell death.

The intrinsic apoptosis pathway can be activated by the release of cytochrome *c*, which is an activator of caspase-9, an initiator caspase, from mitochondria. Active caspase-9 can directly cleave caspase-7, which then directly cleaves PARP more effectively than caspase-3. PARP is cleaved even in the absence of caspase-3 [34]. Finally, cleaved PARP prevents DNA repair during apoptosis [35]. Thus, it was examined whether aviculin (**2**) altered the expression of Bcl-2 and Bax. The expression of Bcl-2 interferes with the release of cytochrome *c*, whereas the expression of Bax induces the release of cytochrome *c* [36]. Treatment with aviculin (**2**) resulted in a decrease in the expression of the anti-apoptotic Bcl-2 protein, along with a concomitant increase in the expression of the pro-apoptotic Bax protein. Together, these data indicate that aviculin (**2**) induced apoptosis in MCF-7 human breast cancer cells via the intrinsic apoptosis pathway. In conclusion, among the nine compounds isolated from the EA fraction of the *L. cuneata* methanolic extract, aviculin (**2**), a lignan glycoside, inhibited metabolic activity in breast cancer cells and induced their apoptosis via an increase in the Bax/Bcl-2 ratio. This apoptosis was accompanied by the activation of caspase-9, caspase-7, and PARP, indicating the induction of the intrinsic apoptosis pathway. Although future studies are necessary to confirm in vitro the effects of aviculin (**2**) on other breast cancer cells with different hormone receptor statuses and in vivo experiments must be performed, the overall results of this study suggest the potential of aviculin (**2**) as a herbal compound for the adjuvant treatment of breast cancer.

## 4. Materials and Methods 

### 4.1. General Experimental Procedures

A Jasco P-1020 polarimeter was used to measure optical rotation. IR spectra were recorded using a Bruker IFS-66/S FT-IR spectrometer (Bruker, Billerica, MA, USA). An Agilent 8453 UV–visible spectrophotometer (Agilent Technologies, Santa Clara, CA, USA) was used to obtain UV spectra. LC/MS analysis was performed using an Agilent 1200 Series HPLC system (Agilent Technologies, Santa Clara, CA, USA) fitted with a diode array detector and a 6130 Series ESI mass spectrometer (Agilent Technologies, Santa Clara, CA, USA) with an analytical Kinetex column (Phenomenex, Torrance, CA, USA) (2.1 mm × 100 mm, 5 μm). A Bruker AVANCE III 800 NMR spectrometer (Bruker, Billerica, MA, USA) operating at 800 MHz (^1^H) and 200 MHz (^13^C) was used to record NMR spectra. Preparative HPLC and semi-preparative HPLC were performed using a Waters 1525 binary HPLC pump and a Waters 996 photodiode array detector. Column chromatography was performed using a silica gel 60 column (Merck, Darmstadt, Germany, 230–400 mesh) and an RP-C18 silica gel column (Merck, Darmstadt, Germany, 230–400 mesh). Molecular sieve column chromatography was performed on a Sephadex LH-20 column (Pharmacia, Uppsala, Sweden). The packing material for open-column chromatography was Diaion HP-20 (Mitsubishi Chemical, Tokyo, Japan). Merck precoated silica gel F254 plates and reversed-phase (RP)-18 F254s plates were used for TLC. TLC pots were detected under UV light or by heating and spraying with anisaldehyde-sulfuric acid.

### 4.2. Plant Material 

The *L. cuneata* samples were collected in October 2016 from Mt. Bangtae, Inje, Kangwon province, Republic of Korea. Prof. K. H. Kim, one of the authors of this paper, verified the material. We deposited a voucher specimen (YKM-2016) at the herbarium of the School of Pharmacy, Sungkyunkwan University, Suwon, Republic of Korea.

### 4.3. Extraction and Isolation 

We extracted 4.2 kg of the desiccated aerial portions of *L. cuneata* at room temperature using 4.2 L of 80% MeOH for 3 days and filtered out the sediment. This extraction process was conducted three times. We then obtained 401.8 g of MeOH extract by rotary evaporation to remove the solvent. The extract was dissolved in 2 L of distilled H_2_O after evaporation and then solvent-fractionized with *n*-hexane, CH_2_Cl_2_, EtOAc, and *n*-BuOH (2 L for each solvent, three times). The weights were 20.6, 0.7, 12.7, and 69.3 g for the *n*-hexane-, CH_2_Cl_2_-, EtOAc-, and *n*-BuOH-soluble fractions, respectively. 

The EtOAc-soluble fraction was separated by Diaion HP-20 column chromatography with a concentration gradient of solvent (MeOH-H_2_O, 0%–20%–40%–60%–80%–100% MeOH) to obtain six fractions (A–F). Six sub-fractions (D_1_–D_6_) were prepared from fraction D using RP-C18 column chromatography (gradient solvent system of MeOH-H_2_O; 30%–100% MeOH). Ten sub-fractions (D_3_1–D_3_10) were prepared from the sub-fraction D_3_ (2.8 g) using silica gel column chromatography (gradient solvent system of CH_2_Cl_2_-MeOH-H_2_O; 15:1:0–9:3:0.5 *v*/*v*/*v*). Compounds **8** (5.1 mg, *t*_R_ = 34.0 min, 0.00127%) and **9** (1.8 mg, *t*_R_ = 36.5 min, 0.00045%) were obtained from sub-fraction D_3_-3 (33.5 mg) by semi-preparative HPLC using a linear gradient solvent system of MeOH (20%–50% in 60 min) with a Phenomenex Luna phenyl-hexyl column (250 mm × 10.0 mm, 10 μm, flow rate: 2 mL/min). Four sub-fractions (D_3_71–D_3_74) were yielded from sub-fraction D_3_7 (1.1 g) using RP-C18 column chromatography (60% MeOH). Sub-fraction D_3_72 (506.7 mg) was separated into five sub-fractions (D_3_721–D_3_725) using silica gel column chromatography (gradient solvent system of CH_2_Cl_2_-MeOH-H_2_O; 10:1:0–1:1:0.25 *v*/*v*/*v*). Ten sub-fractions (D_3_-722A–D_3_-722J) were obtained from sub-fraction D_3_-722 (316.4 mg) using Sephadex LH-20 column chromatography (100% MeOH). Sub-fraction D_3_-722B (24.0 mg) was purified by semi-preparative HPLC (19% MeCN, flow rate: 2 mL/min) using the Phenomenex Luna phenyl-hexyl column to obtain compounds **3** (6.3 mg, *t*_R_ = 29.0 min, 0.00156%) and **4** (2.7 mg, *t*_R_ = 32.5 min, 0.00067%). Nine sub-fractions (D_3_741–D_3_749) were obtained from sub-fraction D_3_74 (127.6 mg) using Sephadex LH-20 column chromatography (100% MeOH). Compounds **5** (3.5 mg, *t*_R_ = 30.5 min, 0.00087%) and **6** (3.5 mg, *t*_R_ = 34.0 min, 0.00087%) were obtained from sub-fraction D_3_-746 (24.9 mg) by semi-preparative HPLC using the Phenomenex Luna phenyl-hexyl column (flow rate: 2 mL/min). Nine sub-fractions (D_3_101–D_3_109) were obtained from sub-fraction D_3_10 (132.7 mg) using Sephadex LH-20 column chromatography (80% MeOH). Compounds **1** (1.5 mg, *t*_R_ = 31.5 min, 0.00037%) and **2** (2.4 mg, *t*_R_ = 36.0 min, 0.00059%) were obtained from sub-fraction D_3_-107 (16.6 mg) by semi-preparative HPLC (38% MeOH, flow rate: 2 mL/min) using the Phenomenex Luna phenyl-hexyl column. Finally, sub-fraction D_3_-109 (17.2 mg) was purified by semi-preparative HPLC (16% MeCN) using the Phenomenex Luna phenyl-hexyl column with a flow rate of 2 mL/min to isolate compound **7** (2.2 mg, *t*_R_ = 27.5 min, 0.00054%).

### 4.4. Cell Culture

The MCF-7 human breast cancer cell line was purchased from the American Type Culture Collection (Manassas, VA, USA). The cells were cultured at 37 °C in a humidified atmosphere with 5% CO_2_ in Roswell Park Memorial Institute 1640 medium (RPMI 1640) (Cellgro, Manassas, VA, USA) supplemented with 10% fetal bovine serum (Gibco BRL, Carlsbad, MD, USA), 100 units/mL penicillin, and 100 mg/mL streptomycin.

### 4.5. Ez-Cytox Assay

Ez-Cytox assay kit (Dail Lab Service Co., Seoul, Korea) was used to measured metabolic activity of cells based on the conversion of the tetrazolium salt WST-1 into a water-soluble formazan by mitochondrial dehydrogenases present in the metabolically active cells. To evaluate the effect of compounds isolated from *L. cuneata*, MCF-7 cells (1 × 10^4^ cells/well) were cultured in 96-well plates for 24 h. The cells were treated with either 0.5% DMSO (control) or the indicated concentrations of sample (*L. cuneata* methanolic extract fractions or isolated compounds **1**–**9**) dissolved in 0.5% DMSO for 24 h. Then, 10 μL of the kit reagent were added to each well and incubated for 30 min. The absorbance of live cells was measured at 450 nm using a microplate reader (PowerWave XS; Bio-Tek Instruments, Winooski, VT, USA). Absorbance of background (culture medium + kit reagent) was subtracted from each group. Metabolic activity was quantified by normalizing the absorbance readings to the cells treated with 0.5% DMSO (control) for each individual group, set at 100% metabolic activity.

### 4.6. Western Blotting Analysis

MCF-7 cells (4 × 10^5^ cells/well) were cultured in 96-well plates for 24 h. The cells were treated with either 0.5% DMSO (control), or the indicated concentrations of sample (*L. cuneata* methanolic extract fractions or isolated compounds **1**–**9**) dissolved in 0.5% DMSO. After 24 h, the cells were prepared in ice-cold radioimmunoprecipitation assay buffer (Cell Signaling Technology, Inc., MA, USA) supplemented with 1× ethylenediaminetetraacetic acid (EDTA)-free protease inhibitor cocktail and 1 mM phenylmethylsulfonyl fluoride (PMSF) on ice in accordance with the manufacturer’s instructions. After the samples were centrifuged (112 *g* for 20 min at 4 °C), the supernatant was collected and the total protein concentration of each extract was determined using a bicinchoninic acid (BCA) protein assay kit in accordance with the manufacturer’s instructions. Equal amounts of proteins (20 μg total protein per lane) were separated by 10% sodium dodecyl sulfate polyacrylamide gel electrophoresis (SDS–PAGE) for 90 min at 110 V and then transferred onto a polyvinylidene difluoride (PVDF) membrane. Non-specific binding to the membrane was blocked by incubation of the membrane in 5% skim milk for 1 h at room temperature. Then, the membrane was incubated with primary antibodies against cleaved caspase-8 (# 9496S, clone 18C8), Bax (# 5023S, clone D2E11), Bcl-2 (# 4223S, clone D55G8), cleaved caspase-9 (# 20750S, clone D8I9E), cleaved caspase-7 (# 8438S, clone D6H1), poly (ADP-ribose) polymerase (PARP) (# 5625S, clone D64E10), and glyceraldehyde 3-phosphate dehydrogenase (GAPDH) (# 5174S, clone D16H11), all purchased from Cell Signaling Technology, Inc. (Danvers, MA, USA), for 1 h at room temperature, followed by incubation with horseradish peroxidase-conjugated rabbit secondary antibody for 1 h at room temperature. The bands were visualized using enhanced chemiluminescence (ECL) Advance Western Blotting Detection Reagents (GE Healthcare; Cambridge, UK) and a FUSION Solo Chemiluminescence System (PEQLAB Biotechnologie GmbH; Erlangen, Germany).

### 4.7. Cell Staining with Annexin V

To detect apoptotic cell death, the annexin V Alexa Fluor 488 binding assay was performed in accordance with the manufacturer’s instructions. The cells were treated with either 0.5% DMSO (control), or the indicated concentrations of sample (*L. cuneata* methanolic extract fractions or isolated compounds **1**–**9**) dissolved in 0.5% DMSO for 24 h, as described in the literatures [36,37,38]. The cells were harvested and prepared to the same concentration of cells for each individual group. The same concentration of approximately 5 × 10^5^ cells/100 μL of annexin binding buffer (Invitrogen; Temecula, CA, USA) was incubated with 5 μL of annexin V Alexa Fluor 488 (Invitrogen; Temecula, CA, USA) for 30 min in a dark at room temperature. The stained cells with annexin V Alexa Fluor 488 were resuspended with an addition of 1 μL of propidium iodide in the 100 μL of annexin binding buffer, and then incubated for 1 min in the dark at room temperature. The percentage of annexin V-positive apoptotic cells was counted in 10 different fields (40× magnification) per slide using the Tali Image-Based Cytometer (Invitrogen; Temecula, CA, USA).

### 4.8. Cell Staining with Hoechst 33342

To detect nuclear condensation, the cell staining with Hoechst 33342 was performed. MCF-7 cells were treated as described above. Two microliters of Hoechst 33342 solution were added to each well. After incubation for 10 min, the stained cells were observed with a fluorescence microscopy.

### 4.9. Statistical Analysis

Data are presented as the mean ± standard deviation (SD). Statistical significance was determined using Kruskal–Wallis test. *p*-values of less than 0.05 were considered to indicate a statistically significant difference. 

## 5. Conclusions

Our study demonstrated aviculin isolated from the *L. cuneata* methanolic extract induced apoptosis in MCF-7 human breast cancer cells. Aviculin inhibited the metabolic activity in MCF-7 cells in a concentration-dependent manner, with an IC_50_ value of 75.47 ± 2.23 μM. Aviculin induced apoptotic cell death through the intrinsic apoptosis pathway, by upregulating the expression of initiator caspase-9, executioner caspase-7, and poly (ADP-ribose) polymerase and increasing the Bax/Bcl-2 ratio. These findings suggested that the apoptotic effect of aviculin on breast cancer cells was mediated by the intrinsic apoptosis pathway. Our findings provide experimental evidence to suggest that aviculin may be a potential lead compound for breast cancer therapy. 

## Supplementary Materials

The following are available online, Figure S1: ^1^H-NMR data of compound **1**; Figure S2: ^1^H-NMR data of compound **2**; Figure S3: ^1^H-NMR data of compound **3**; Figure S4: ^1^H-NMR data of compound **4**; Figure S5: ^1^H-NMR data of compound **5**; Figure S6: ^1^H-NMR data of compound **6**; Figure S7: ^1^H-NMR data of compound **7**; Figure S8: ^1^H-NMR data of compound **8**; Figure S9: ^1^H-NMR data of compound **9**; Figure S10: LC/MS analysis for purity of compounds **1**–**9**.

## References

[1] Hwang E.S., Nho J.H. (2019). Lifestyle Intervention for Breast Cancer Women. J. Lifestyle Med..

[2] Nizioł M., Kostrzewska B., Kamińska D., Domurat M., Zińczuk J., Misiura M., Guzińska-Ustymowicz K., Pryczynicz A. (2019). Symptoms of colorectal cancer contributes to its localization and advancement. Prog. Health Sci..

[3] Neugut A.I., Hillyer G.C., Kushi L.H., Lamerato L., Nathanson S.D., Ambrosone C.B., Bovbjerg D.H., Mandelblatt J.S., Magai C., Tsai W.Y. (2012). The Breast Cancer Quality of Care Study (BQUAL): A Multi-Center Study to Determine Causes for Noncompliance with Breast Cancer Adjuvant Therapy. Breast J..

[4] Kim M.S., Sharma B.R., Rhyu D.Y. (2016). Beneficial effect of *Lespedeza cuneata* (G. Don) water extract on streptozotocin-induced type 1 diabetes and cytokine-induced beta-cell damage. Nat. Prod. Sci..

[5] Baek J., Lee D., Lee T.K., Song J.H., Lee J.S., Lee S., Yoo S.W., Kang K.S., Moon E., Lee S. (2018). (−)-9′-O-(α-l-Rhamnopyranosyl) lyoniresinol from *Lespedeza cuneata* suppresses ovarian cancer cell proliferation through induction of apoptosis. Bioorg. Med. Chem. Lett..

[6] Seong J.S., Xuan S.H., Park S.H., Lee K.S., Park Y.M., Park S.N. (2017). Antioxidative and antiaging activities and component analysis of *Lespedeza cuneata* G. Don extracts fermented with *Lactobacillus pentosus*. J. Microbiol. Biotechnol..

[7] Lee J., Ji J., Park S.H. (2018). Antiwrinkle and antimelanogenesis activity of the ethanol extracts of *Lespedeza cuneata* G. Don for development of the cosmeceutical ingredients. Food Sci. Nutr..

[8] Park B.K., Kim C.W., Kwon J.E., Negi M., Koo Y.T., Lee S.H., Baek D.H., Noh Y.H., Kang S.C. (2019). Effects of *Lespedeza cuneata* aqueous extract on testosterone-induced prostatic hyperplasia. Pharm. Biol..

[9] Park B., Kwon J.E., Cho S.M., Kim C.W., Koo Y.T., Lee S.H., Lee H.M., Kang S.C. (2018). Protective effect of *Lespedeza cuneata* ethanol extract on Bisphenol A-induced testicular dysfunction in vivo and in vitro. Biomed. Pharmacother..

[10] Matsuura S., Iinuma M., Ito E., Takami H., Kagei K. (1978). Studies on the constituents of the useful plants. VIII. The constituents of *Lespedeza cuneata* G. Don (author’s transl). Yakugaku Zasshi.

[11] Yoo G., Park S.J., Lee T.H., Yang H., Baek Y.S., Kim N., Kim Y.J., Kim S.H. (2015). Flavonoids isolated from *Lespedeza cuneata* G. Don and their inhibitory effects on nitric oxide production in lipopolysaccharide-stimulated BV-2 microglia cells. Pharmacogn. Mag..

[12] Zhang C.F., Zhou J., Yang J.Z., Li C.J., Ma J., Zhang D., Li L., Zhang D.M. (2016). Three new lignanosides from the aerial parts of *Lespedeza cuneata*. J. Asian Nat. Prod. Res..

[13] So H.M., Eom H.J., Lee D., Kim S., Kang K.S., Lee I.K., Baek K.H., Park J.Y., Kim K.H. (2018). Bioactivity evaluations of betulin identified from the bark of *Betula platyphylla* var. *japonica* for cancer therapy. Arch. Pharmacal Res..

[14] Yu J.S., Roh H.S., Baek K.H., Lee S., Kim S., So H.M., Moon E., Pang C., Jang T.S., Kim K.H. (2018). Bioactivity-guided isolation of ginsenosides from Korean Red Ginseng with cytotoxic activity against human lung adenocarcinoma cells. J. Ginseng Res..

[15] Baek S.C., Choi E., Eom H.J., Jo M.S., Kim S., So H.M., Kim S.H., Kang K.S., Kim K.H. (2018). LC/MS-based analysis of bioactive compounds from the bark of *Betula platyphylla* var. *japonica* and their effects on regulation of adipocyte and osteoblast differentiation. Nat. Prod. Sci..

[16] Lee S., Lee S., Roh H.S., Song S.S., Ryoo R., Pang C., Baek K.H., Kim K.H. (2018). Cytotoxic constituents from the sclerotia of *Poria cocos* against human lung adenocarcinoma cells by inducing mitochondrial apoptosis. Cells.

[17] Trinh T.A., Park E.J., Lee D., Song J.H., Lee H.L., Kim K.H., Kim Y., Jung K., Kang K.S., Yoo J.E. (2019). Estrogenic Activity of Sanguiin H-6 through Activation of Estrogen Receptor α Coactivator-binding Site. Nat. Prod. Sci..

[18] Baek J., Lee T., Song J.H., Choi E., Ko H.J., Lee S., Choi S., Lee S., Yoo S.W., Kim S.H. (2018). Lignan Glycosides and Flavonoid Glycosides from the Aerial Portion of *Lespedeza cuneata* and Their Biological Evaluations. Molecules.

[19] Zhou J., Li C.J., Yang J.Z., Ma J., Wu L.Q., Wang W.J., Zhang D.M. (2016). Phenylpropanoid and lignan glycosides from the aerial parts of *Lespedeza cuneata*. Phytochemistry.

[20] Kaneda N., Kinghorn A.D., Farnsworth N.R., Tuchinda P., Udchachon J., Santisuki T., Reutrakul V. (1990). Two diarylheptanoids and a lignan from *Casuarina junghuhniana*. Phytochemistry.

[21] Ochung A.A., Manguro L.A.O., Owuor P.O., Jondiko I.O., Nyunja R.A., Akala H., Mwinzi P., Opiyo S.A. (2015). Bioactive carbazole alkaloids from *Alysicarpus ovalifolius* (Schumach). J. Korean Soc. Appl. Boil. Chem..

[22] Kazuma K., Noda N., Suzuki M. (2003). Malonylated flavonol glycosides from the petals of *Clitoria ternatea*. Phytochemistry.

[23] Rayyan S., Fossen T., Nateland H.S., Anderson O.M. (2005). Isolation and identification of flavonoids, including flavone rotamers, from the herbal drug ’Crataegi folium cum flore’ (hawthorn). Phytochem. Anal..

[24] Feng W.S., Li K.K., Zheng X.K. (2011). A new norlignan lignanoside from *Selaginella moellendorffii* Hieron. Acta Pharm. Sin..

[25] Phadungkit M., Luanratana O. (2006). Anti-*Salmonella* activity of constituents of *Ardisia elliptica* Thunb. Nat. Prod. Res..

[26] Ionkova I. (2011). Anticancer lignans-from discovery to biotechnology. Mini-Rev. Med. Chem..

[27] Ionkova I. (2010). Biotechnology and modern production of plant made pharmaceuticals: Anticancer compounds. Int. J. Curr. Chem..

[28] Imbert T. (1998). Discovery of podophyllotoxins. Biochimie.

[29] Ohashi K., Winarno H., Mukai M., Inoue M., Prana M.S., Simanjuntak P., Shibuya H. (2003). Indonesian medicinal plants. XXV. Cancer cell invasion inhibitory effects of chemical constituents in the parasitic plant *Scurrula atropurpurea* (Loranthaceae). Chem. Pharm. Bull..

[30] Elmore S. (2007). Apoptosis: A review of programmed cell death. Toxicol. Pathol..

[31] Melino G., Knight R., Nicotera P. (2005). How many ways to die? How many different models of cell death?. Cell Death Differ..

[32] Kumar R., Herbert P., Warrens A. (2005). An introduction to death receptors in apoptosis. Int. J. Surg..

[33] Szabo C. (2000). Cell Death: The Role of PARP.

[34] Soldani C., Scovassi A. (2002). Poly (ADP-ribose) polymerase-1 cleavage during apoptosis: An update. Apoptosis.

[35] Germain M., Affar E.B., D’Amours D., Dixit V.M., Salvesen G.S., Poirier G.G. (1999). Cleavage of automodified poly (ADP-ribose) polymerase during apoptosis evidence for involvement of caspase-7. J. Biol. Chem..

[36] Rossé T., Olivier R., Monney L., Rager M., Conus S., Fellay I., Jansen B., Borner C. (1998). Bcl-2 prolongs cell survival after Bax-induced release of cytochrome c. Nature.

[37] Ahn S.Y., Jo M.S., Lee D., Baek S.E., Baek J., Yu J.S., Jo J., Yun H., Kang K.S., Yoo J.E. (2019). Dual effects of isoflavonoids from *Pueraria lobata* roots on estrogenic activity and anti-proliferation of MCF-7 human breast carcinoma cells. Bioorg. Chem..

[38] Lee D., Lee D.S., Jung K., Hwang G.S., Lee H.L., Yamabe N., Lee H.J., Eom D.W., Kim K.H., Kang K.S. (2018). Protective effect of ginsenoside Rb1 against tacrolimus-induced apoptosis in renal proximal tubular LLC-PK1 cells. J. Ginseng Res..

