# Supplementary material for: Aviculin Isolated from Lespedeza cuneata Induce Apoptosis in Breast Cancer Cells through Mitochondria-Mediated Caspase Activation Pathway
Source: Molecules. 2020 Apr 8;25(7):1708. doi: 10.3390/molecules25071708 (PMC7180549; doi:10.3390/molecules25071708)
Supplement: Supplementary file 1 [file molecules-25-01708-s001.pdf]

# Supplementary Materials

# Aviculin Isolated from *Lespedeza cuneata* Induce Apoptosis in Breast Cancer Cells through Mitochondria-Mediated Caspase Activation Pathway

**Dahae Lee [1,†], Yong Hoon Lee [2,†], Kwang Ho Lee [2,†], Bum Soo Lee [2], Akida Alishir [2], Yoon-Joo Ko [3], Ki Sung Kang [1,*] and Ki Hyun Kim [2,*]**

[1] College of Korean Medicine, Gachon University, Seongnam 13120, Korea; pjsldh@naver.com
[2] School of Pharmacy, Sungkyunkwan University, Suwon 16419, Korea; yhl2090@naver.com (Y.H.L.); sholaly@naver.com (K.H.L.); kosboybs@naver.com (B.S.L.); akida.alishir@gmail.com (A.A.)
[3] Laboratory of Nuclear Magnetic Resonance, National Center for Inter-University Research Facilities (NCIRF), Seoul National University, Gwanak-gu, Seoul 08826, Korea; yjko@snu.ac.kr
* Correspondence: kkang@gachon.ac.kr (K.S.K.); khkim83@skku.edu (K.H.K.); Tel.: +82-31-750-5402 (K.S.K.); +82-31-290-7700 (K.H.K.)
† These authors contributed equally to this work

**Figure S1.** $^{1}$H-NMR data of compound **1**.

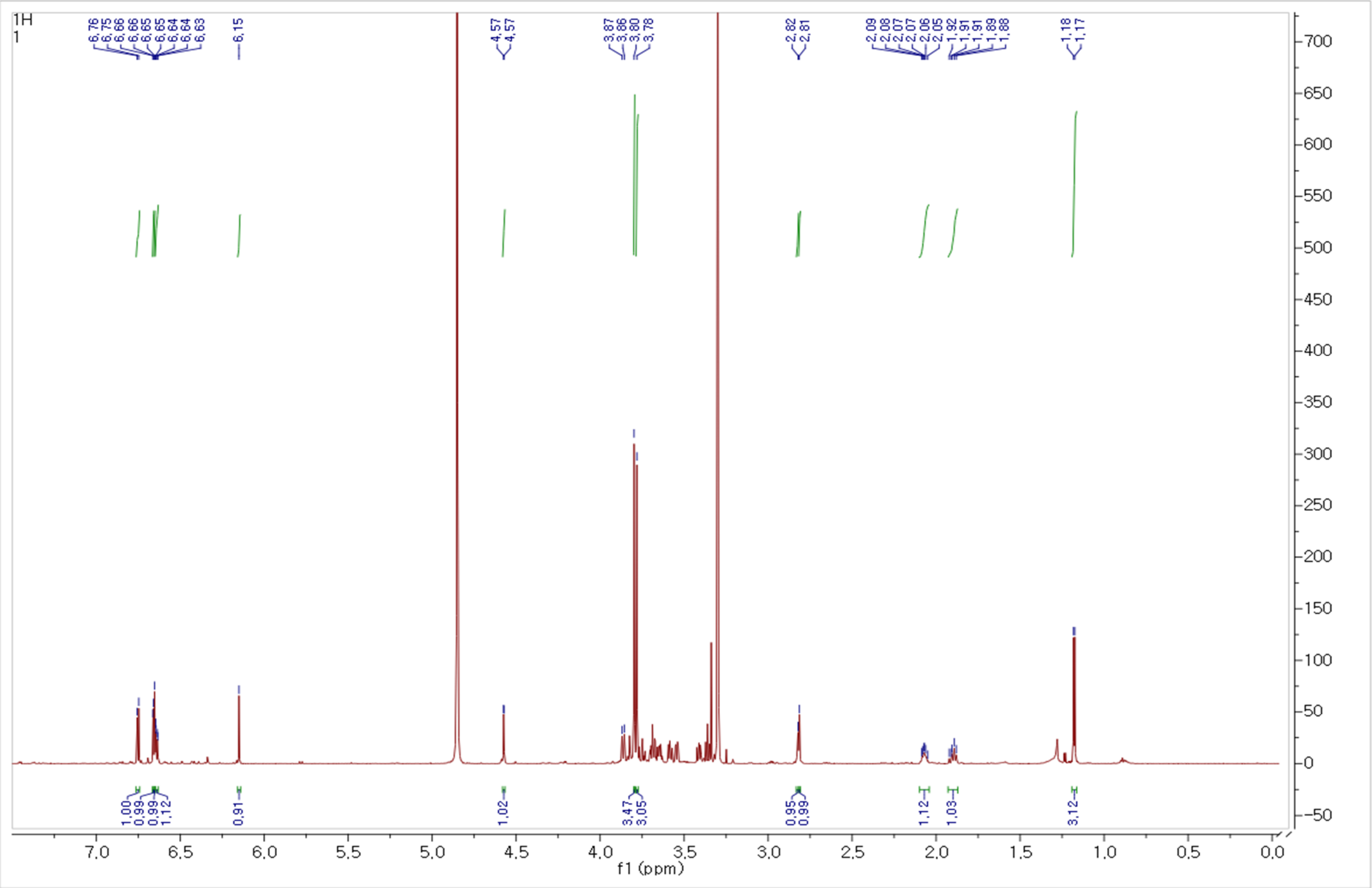

**Figure S2.** $^{1}$H-NMR data of compound **2**.

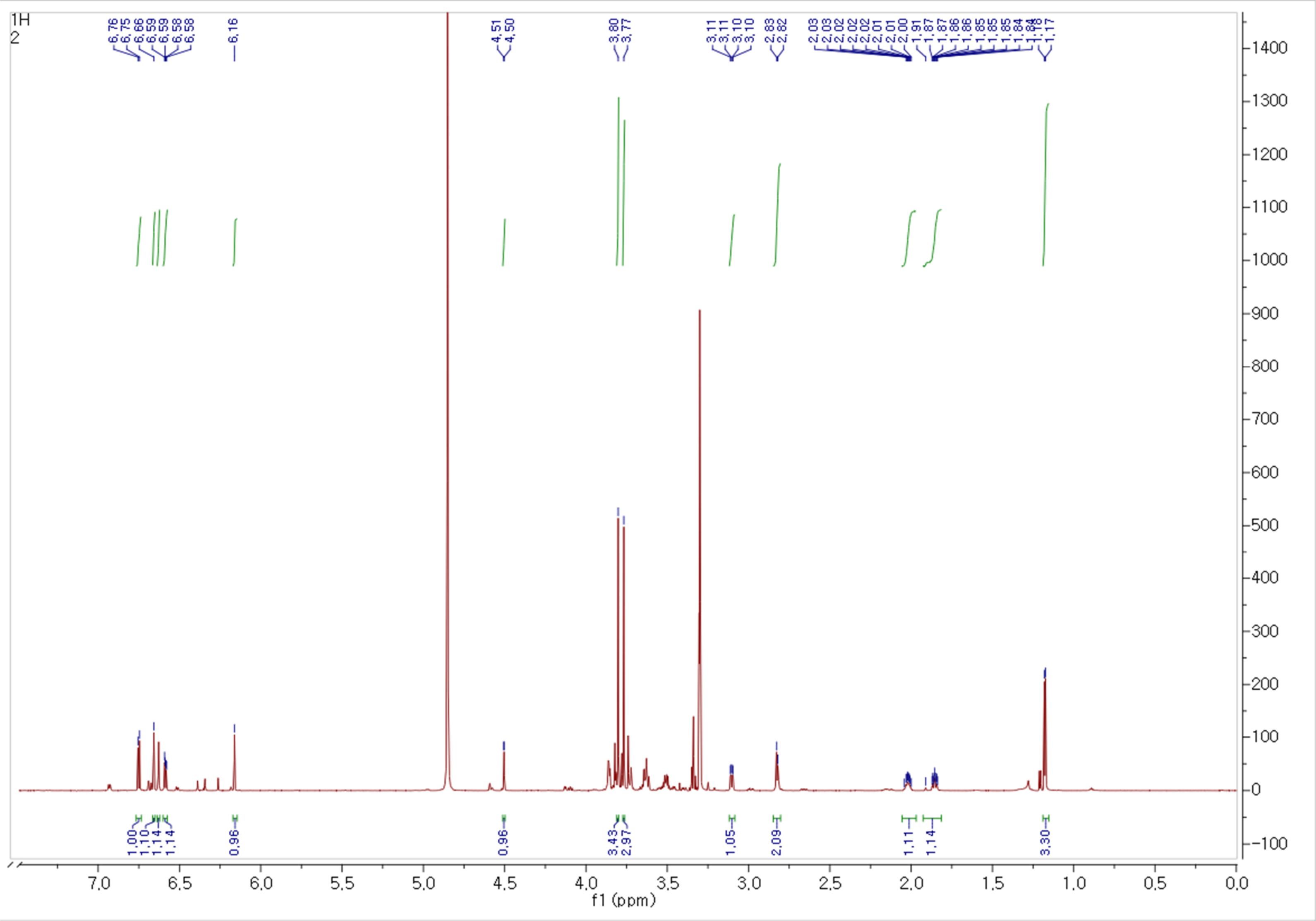

**Figure S3.** $^{1}$H-NMR data of compound **3**.

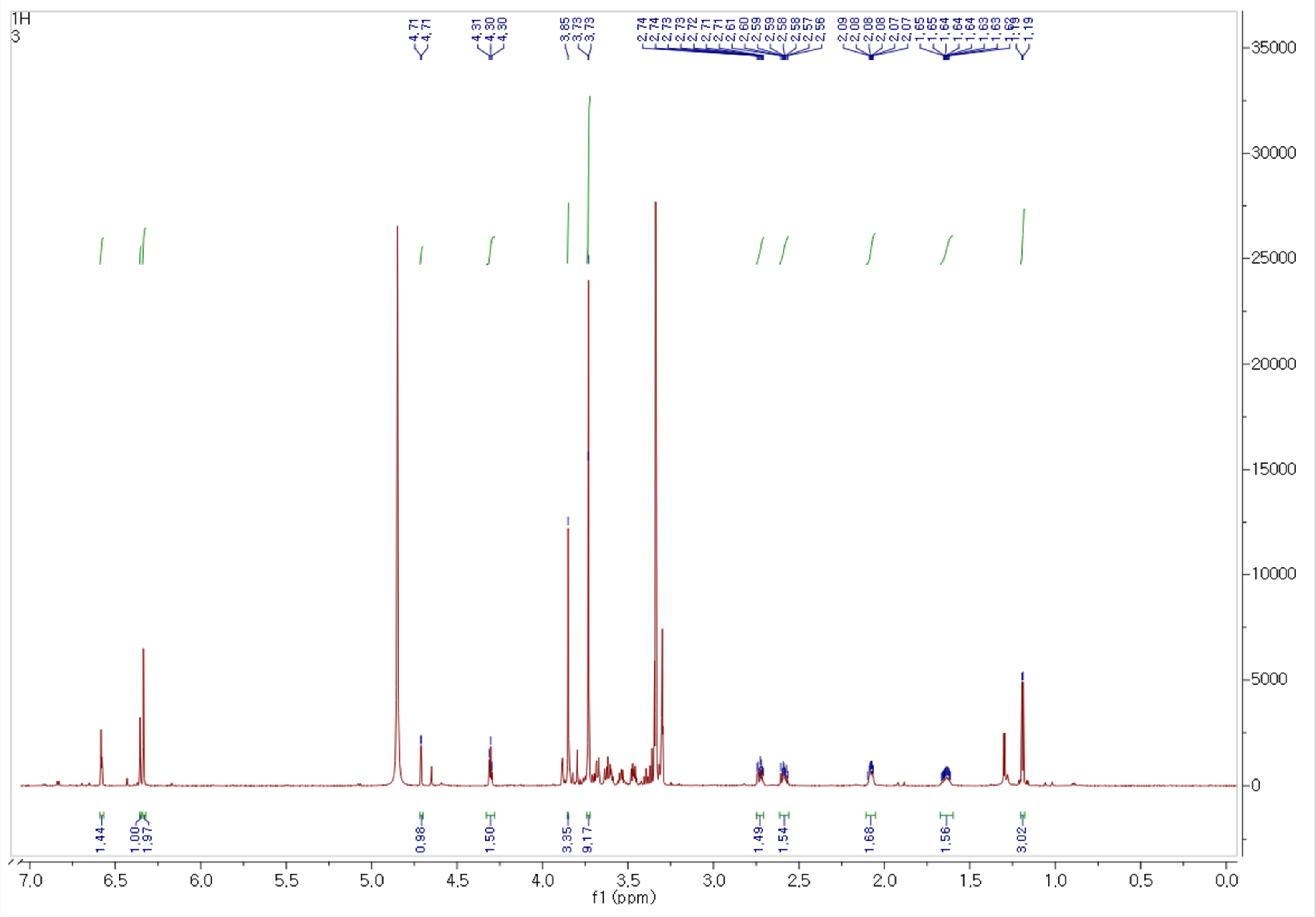

**Figure S4.** $^{1}$H-NMR data of compound **4**.

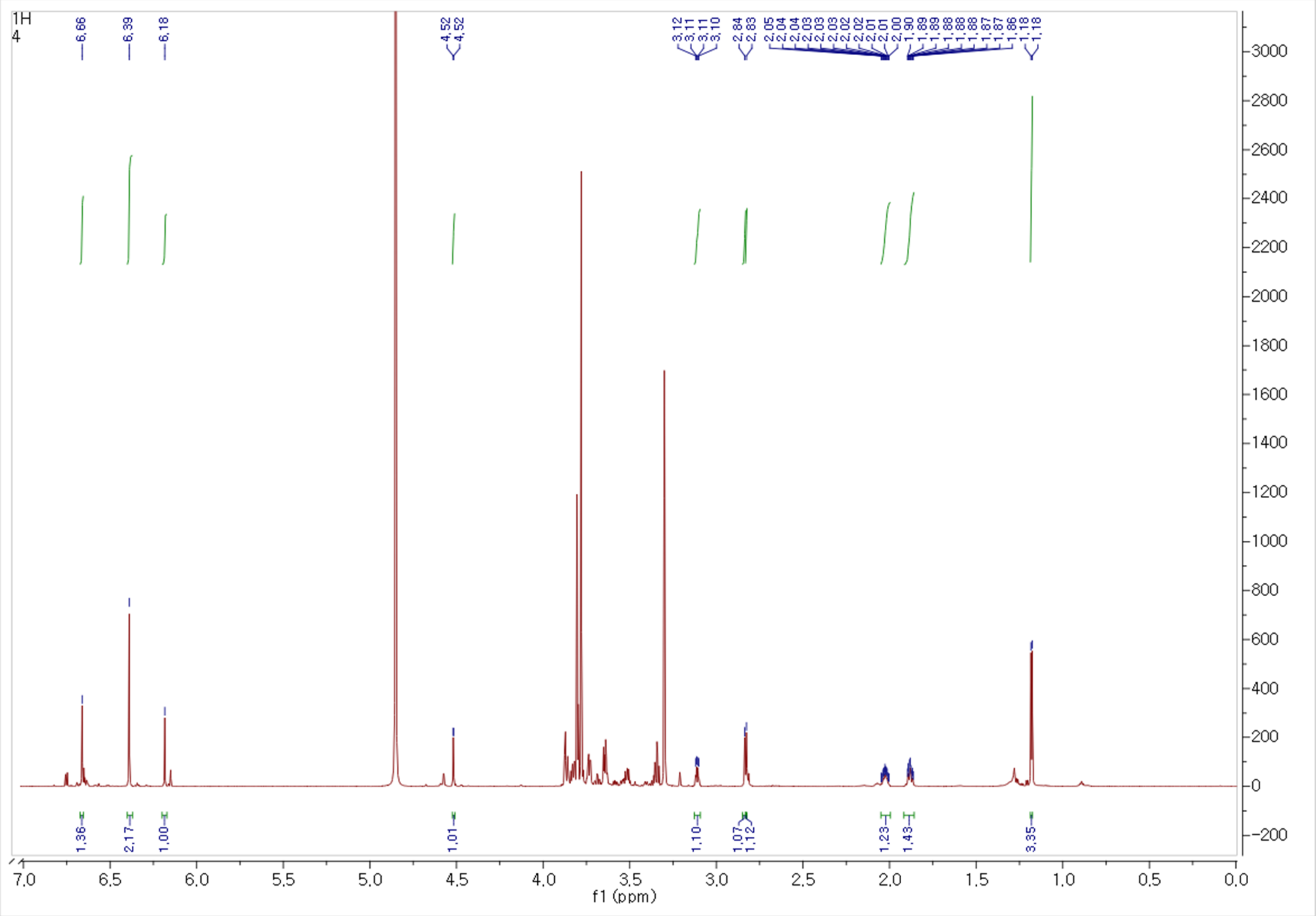

**Figure S5.** $^{1}$H-NMR data of compound **5**.

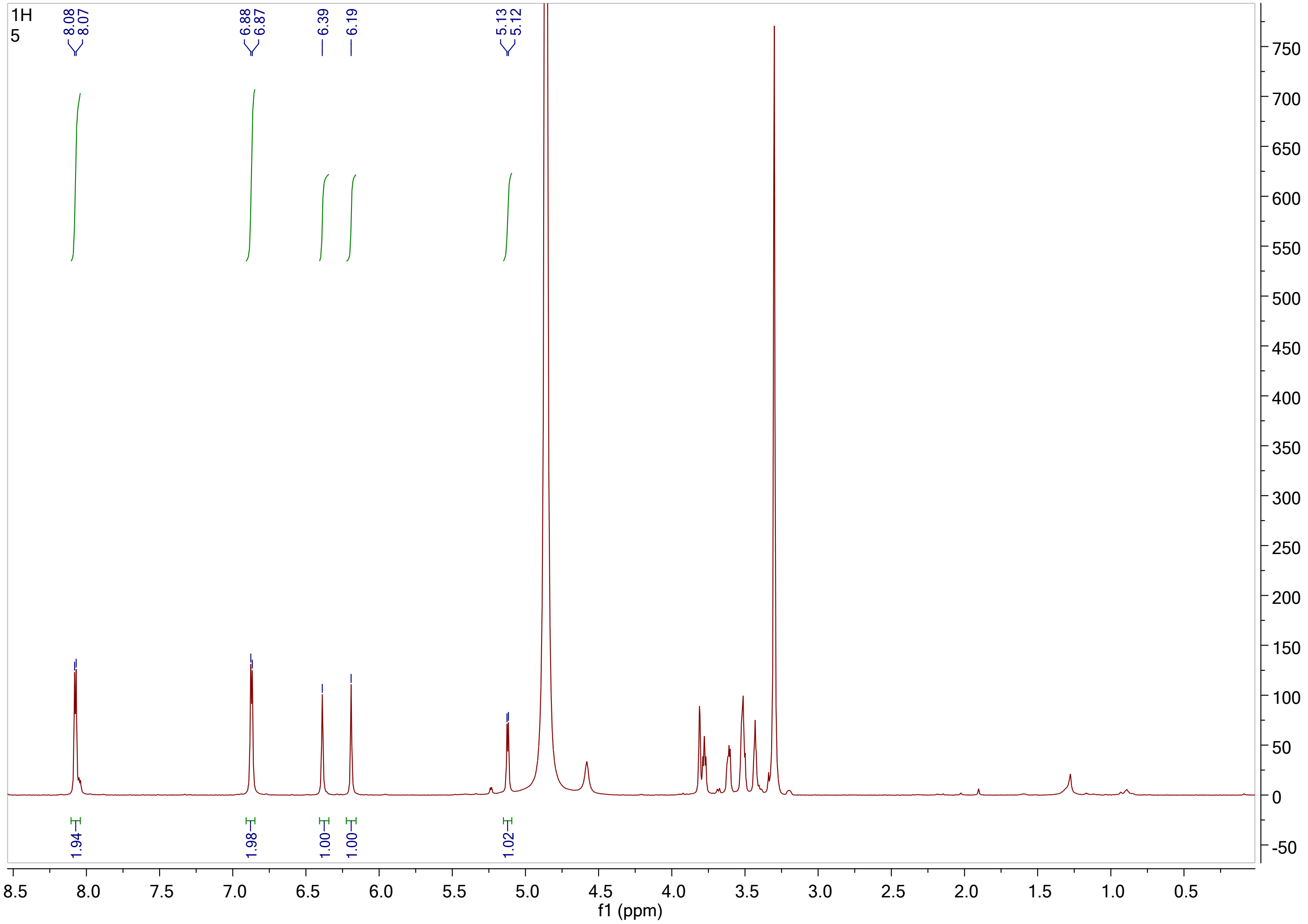

**Figure S6.** $^1$H-NMR data of compound **6**.

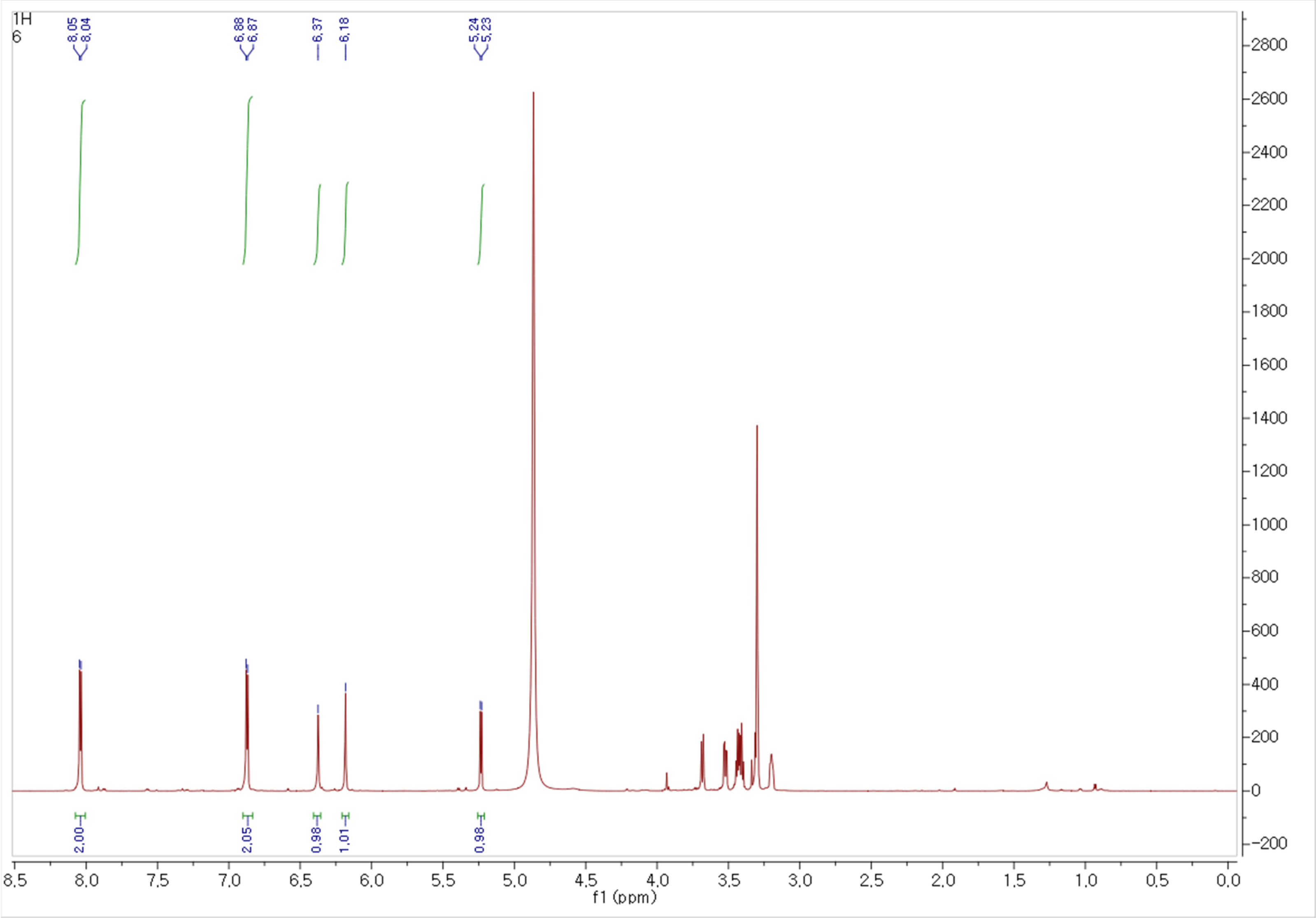

**Figure S7.** $^{1}$H-NMR data of compound **7**.

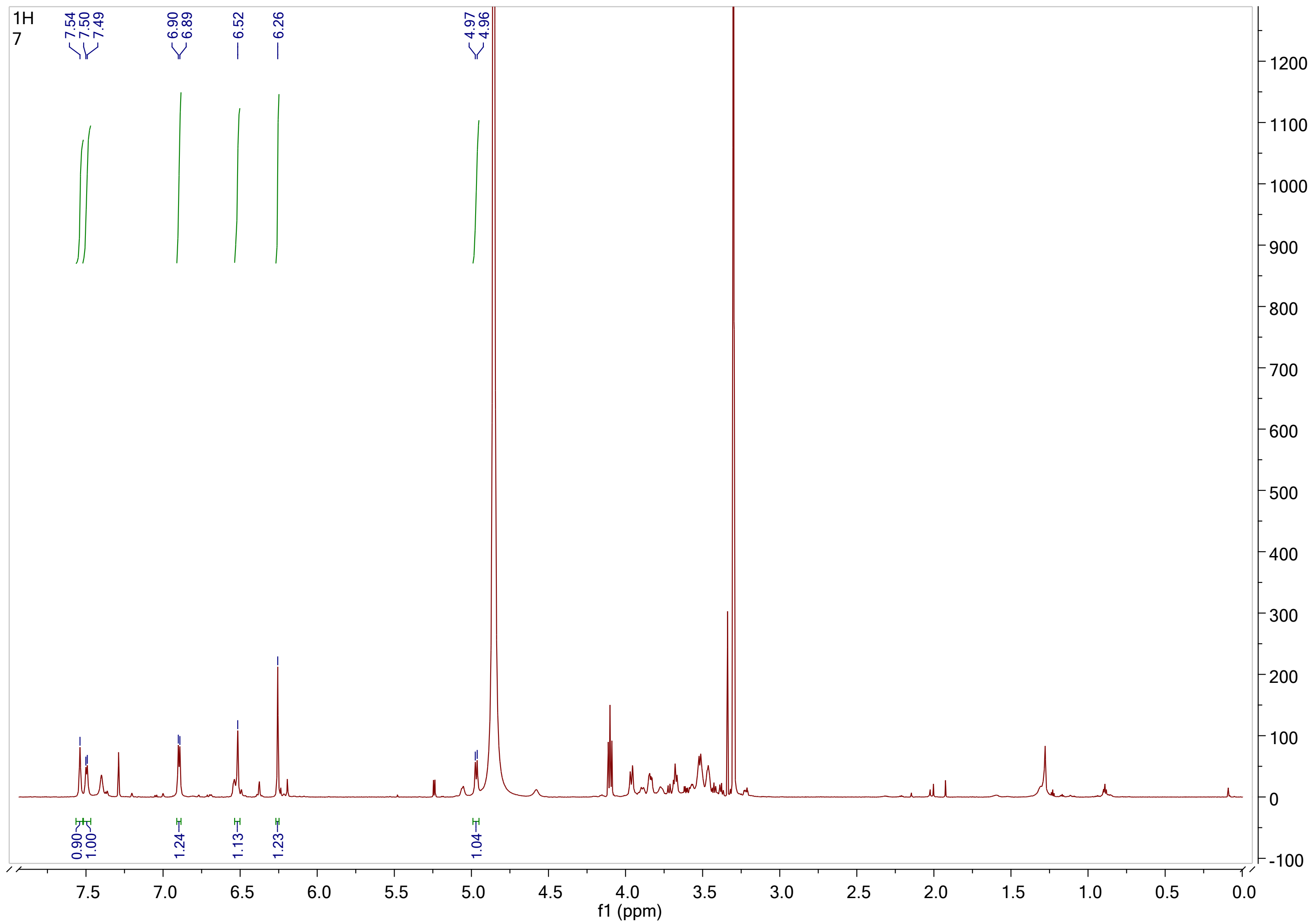

**Figure S8.** $^{1}$H-NMR data of compound **8**.

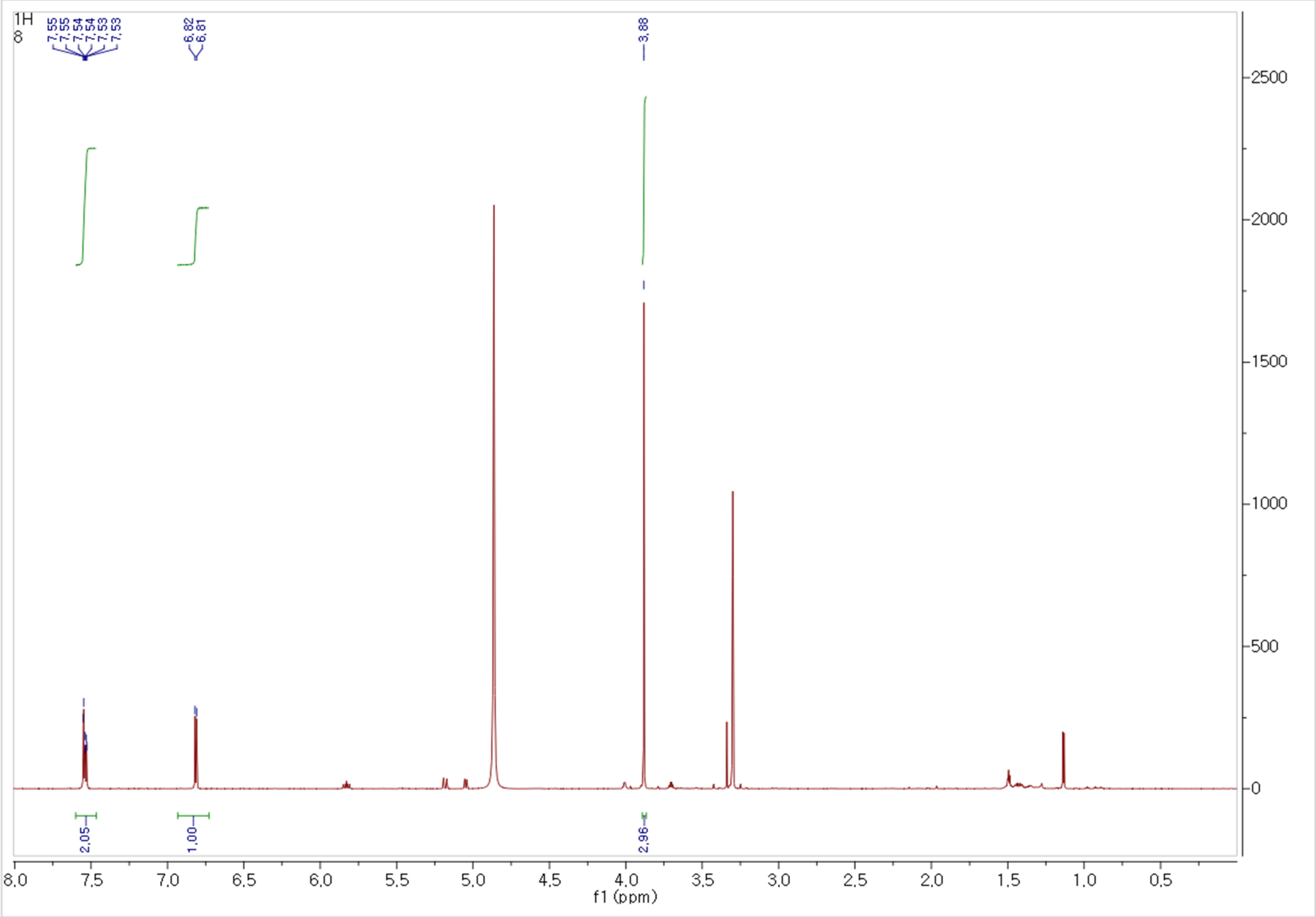

**Figure S9.** $^{1}$H-NMR data of compound **9**.

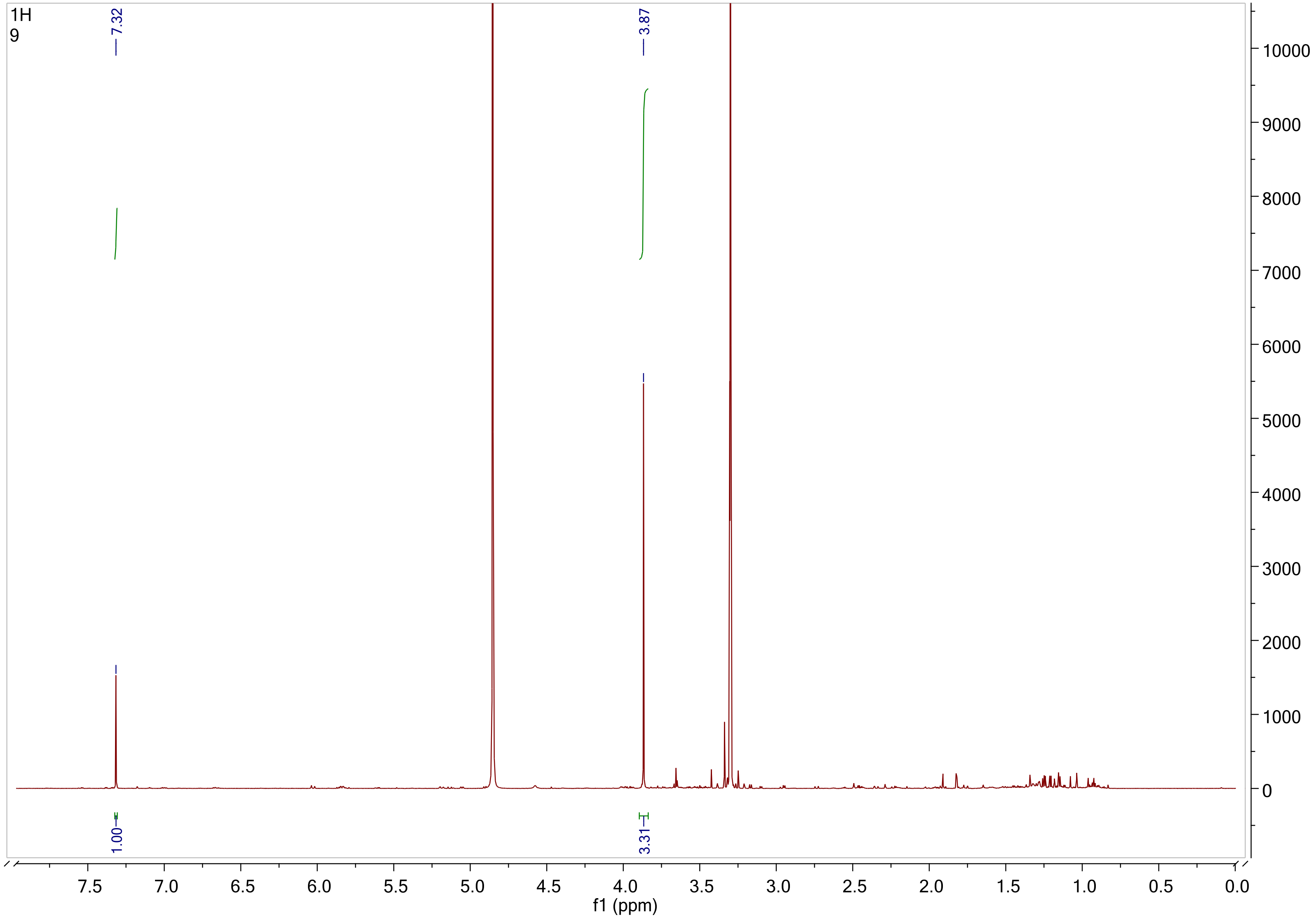

**Figure S10.** LC/MS analysis for purity of compounds **1-9**.

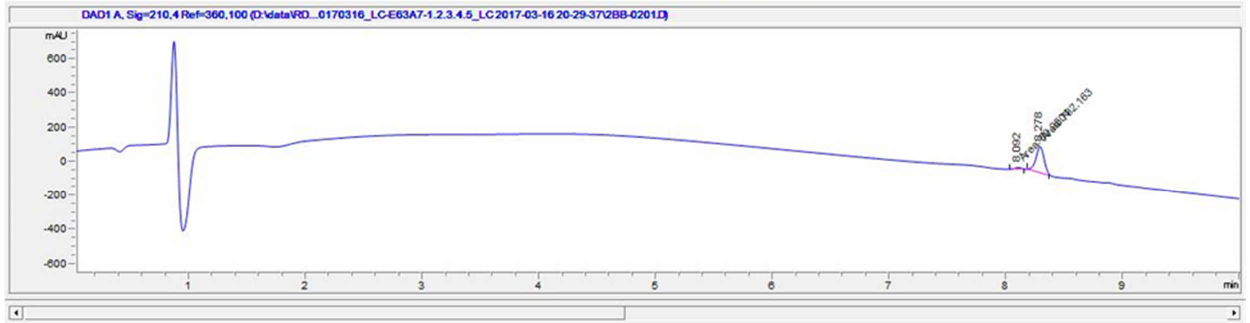


File Information

| | |
|---|---|
| LC-File | 2BB-0201.D |
| File Path | D:\data\RD\[LC]\20170316_LC-E63A7-1.2.3.4.5_LC 2017-03-16 20 |
| Date | 16-Mar-17, 20:51:25 |
| Sample | LC-E63A7-2 |
| Sample Info | |
| Barcode | |
| Operator | SYSTEM |
| Method | 10%~90%MeOH_COMPOUNDS.M |

| # | Time | Area | Height | Width | Area% | Symmetry |
|---|---|---|---|---|---|---|
| 1 | 8.092 | 61 | 13 | 0.0782 | 7.685 | 0.952 |
| 2 | 8.278 | 732.2 | 153.1 | 0.0797 | 92.315 | 0.978 |

**Purity : 92.315%**

Conditions for LC-MS/MS analysis

- Agilent Technologies 1200s
- Column: Phenomenex Kinetex 5 μm C18 100A 100 x 2.1 mm
- Flow: 0.3 mL/min
- Detector: PDA detector (210 nm)
- Mobile phase: Water-MeOH gradient

| Time | MeOH |
|---|---|
| 0.0 | 10 |
| 10 | 90 |

**Compound 1**

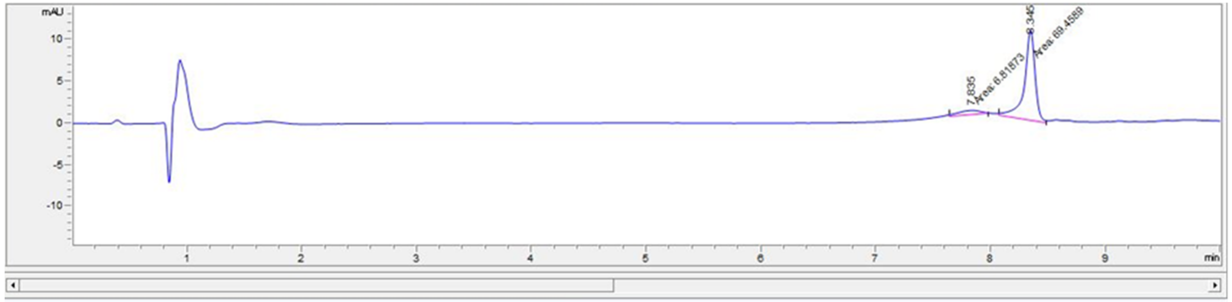


**File Information**

| | |
|---|---|
| LC-File | 2DD-0301.D |
| File Path | D:\data\RD\[LC]\20170306_LC-E63725-1.2.3_LC 2017-03-06 17-0( |
| Date | 06-Mar-17, 17:48:55 |
| Sample | LC-E63725-3 |
| Sample Info | |
| Barcode | |
| Operator | SYSTEM |
| Method | 10%~90%MeOH_COMPOUNDS.M |

| # | Time | Area | Height | Width | Area% | Symmetry |
|---|---|---|---|---|---|---|
| 1 | 7.835 | 6.8 | 5E-1 | 0.2281 | 8.939 | 1.882 |
| 2 | 8.345 | 69.5 | 10.8 | 0.1072 | 91.061 | 1.342 |

Conditions for LC-MS/MS analysis

- Agilent Technologies 1200s
- Column: Phenomenex Kinetex 5 μm C18 100A 100 x 2.1 mm
- Flow: 0.3 mL/min
- Detector: PDA detector (315 nm)
- Mobile phase: Water-MeOH gradient

| Time | MeOH |
|---|---|
| 0.0 | 10 |
| 10 | 90 |

**Purity : 91.061%**

## Compound 2

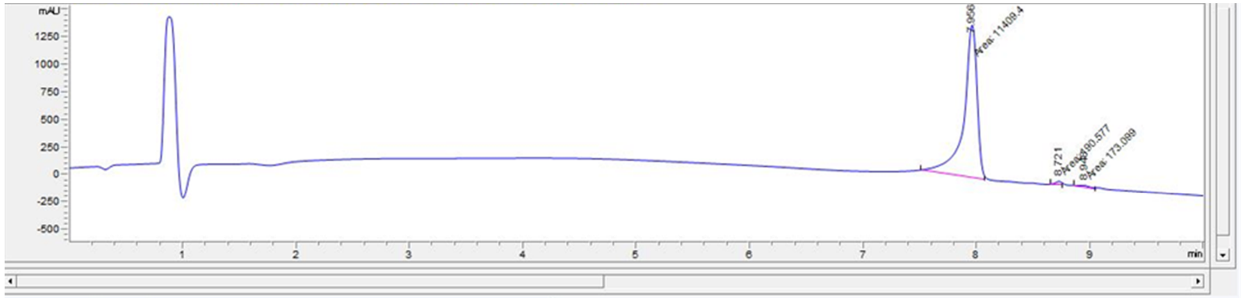


Conditions for LC-MS/MS analysis
- Agilent Technologies 1200s
- Column: Phenomenex Kinetex 5 μm C18 100A 100 x 2.1 mm
- Flow: 0.3 mL/min
- Detector: PDA detector (210 nm)
- Mobile phase: Water-MeOH gradient

**File Information**

| | |
|---|---|
| LC-File | 1FC-0301.D |
| File Path | D:\data\RD\[LC]\20171113_LC-E638221-1~7_LC 2017-11-13 22-0[illegible] |
| Date | 13-Nov-17, 22:49:53 |
| Sample | LC-E638221-3 |
| Sample Info | |
| Barcode | |
| Operator | SYSTEM |
| Method | 10%~90%MeOH_COMPOUNDS.M |

| # | Time | Area | Height | Width | Area% | Symmetry |
|---|---|---|---|---|---|---|
| 1 | 7.956 | 11409.4 | 1390.9 | 0.1367 | 96.911 | 1.69 |
| 2 | 8.721 | 190.6 | 39.7 | 0.08 | 1.619 | 1.334 |
| 3 | 8.948 | 173.1 | 22 | 0.131 | 1.470 | 0.784 |

| Time | MeOH |
|---|---|
| 0.0 | 10 |
| 10 | 90 |

**Purity : 96.911%**

**Compound 3**

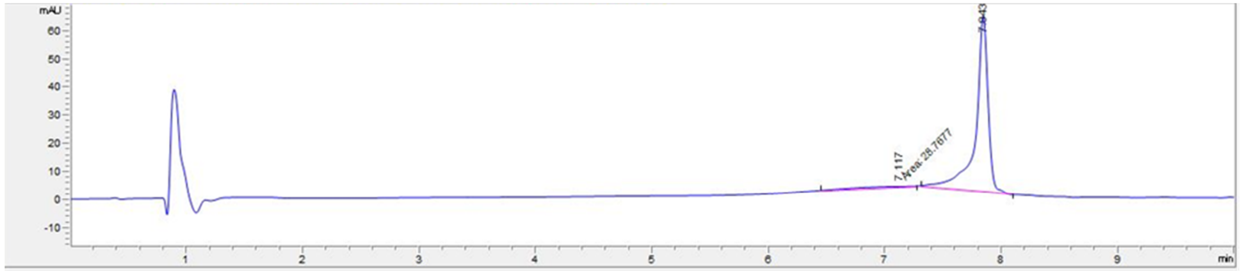


**File Information**

| | |
|---|---|
| LC-File | 2FG-0201.D |
| File Path | D:\data\RD\[LC]\20170428_LC-E637222-1.2.3_LC 2017-04-28 18-: |
| Date | 28-Apr-17, 18:53:03 |
| Sample | LC-E637222-2 |
| Sample Info | |
| Barcode | |
| Operator | SYSTEM |
| Method | 10%~90%MeOH_COMPOUNDS.M |

| # | Time | Area | Height | Width | Area% | Symmetry |
|---|---|---|---|---|---|---|
| 1 | 7.117 | 28.8 | 6.7E-1 | 0.5359 | 5.706 | 3.867 |
| 2 | 7.843 | 475.4 | 62.6 | 0.1086 | 94.294 | 1.695 |

**Purity : 94.294%**

Conditions for LC-MS/MS analysis
- Agilent Technologies 1200s
- Column: Phenomenex Kinetex 5 μm C18 100A 100 x 2.1 mm
- Flow: 0.3 mL/min
- Detector: PDA detector (254 nm)
- Mobile phase: Water-MeOH gradient

| Time | MeOH |
|---|---|
| 0.0 | 10 |
| 10 | 90 |

**Compound 4**

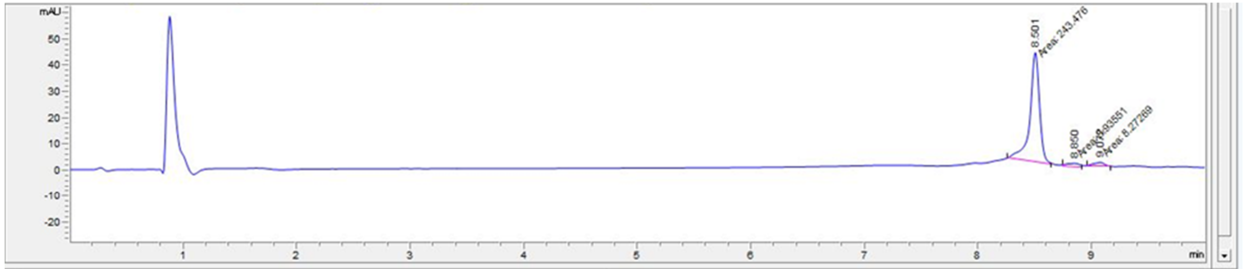


**File Information**

| | |
|---|---|
| LC-File | 1FF-0601.D |
| File Path | D:\data\RD\[LC]\20171113_LC-E638221-1~7_LC 2017-11-13 22-0[illegible] |
| Date | 13-Nov-17, 23:53:02 |
| Sample | LC-E638221-6 |
| Sample Info | |
| Barcode | |
| Operator | SYSTEM |
| Method | 10%~90%MeOH_COMPOUNDS.M |

| # | Time | Area | Height | Width | Area% | Symmetry |
|---|---|---|---|---|---|---|
| 1 | 8.501 | 243.5 | 41.6 | 0.0974 | 93.399 | 1.259 |
| 2 | 8.85 | 8.9 | 1.2 | 0.1243 | 3.428 | 1.271 |
| 3 | 9.072 | 8.3 | 1.3 | 0.1045 | 3.173 | 1.622 |

Conditions for LC-MS/MS analysis
- Agilent Technologies 1200s
- Column: Phenomenex Kinetex 5 µm C18 100A 100 x 2.1 mm
- Flow: 0.3 mL/min
- Detector: PDA detector (254 nm)
- Mobile phase: Water-MeOH gradient

| Time | MeOH |
|---|---|
| 0.0 | 10 |
| 10 | 90 |

**Purity : 93.399%**

**Compound 5**

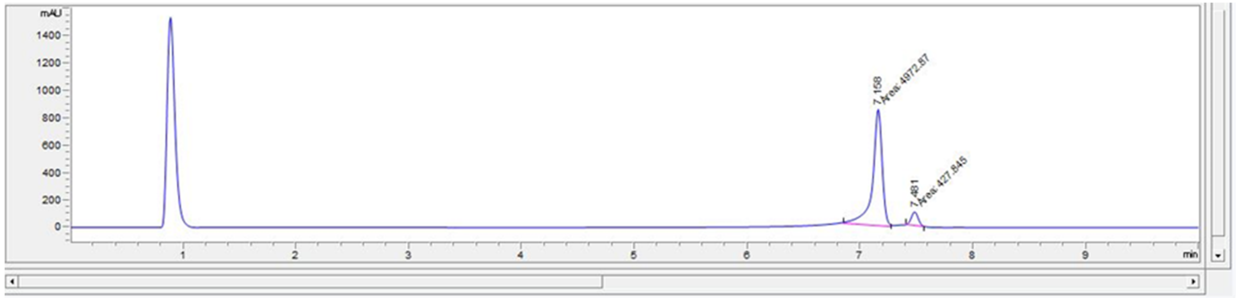


**File Information**

| | |
|---|---|
| LC-File | 1AA-0101.D |
| File Path | D:\data\RD\[LC]\20171030_LC-E638231-1.2.3.4_LC-E63932_LC 20 |
| Date | 30-Oct-17, 17:10:18 |
| Sample | LC-E638231-1 |
| Sample Info | |
| Barcode | |
| Operator | SYSTEM |
| Method | 10%~90%MeOH_COMPOUNDS.M |

| # | Time | Area | Height | Width | Area% | Symmetry |
|---|---|---|---|---|---|---|
| 1 | 7.158 | 4972.9 | 848.3 | 0.0977 | 92.078 | 1.409 |
| 2 | 7.481 | 427.8 | 100.2 | 0.0712 | 7.922 | 0.958 |

Conditions for LC-MS/MS analysis
- Agilent Technologies 1200s
- Column: Phenomenex Kinetex 5 μm C18 100A 100 x 2.1 mm
- Flow: 0.3 mL/min
- Detector: PDA detector (254 nm)
- Mobile phase: Water-MeOH gradient

| Time | MeOH |
|---|---|
| 0.0 | 10 |
| 10 | 90 |

**Purity : 92.078%**

**Compound 6**

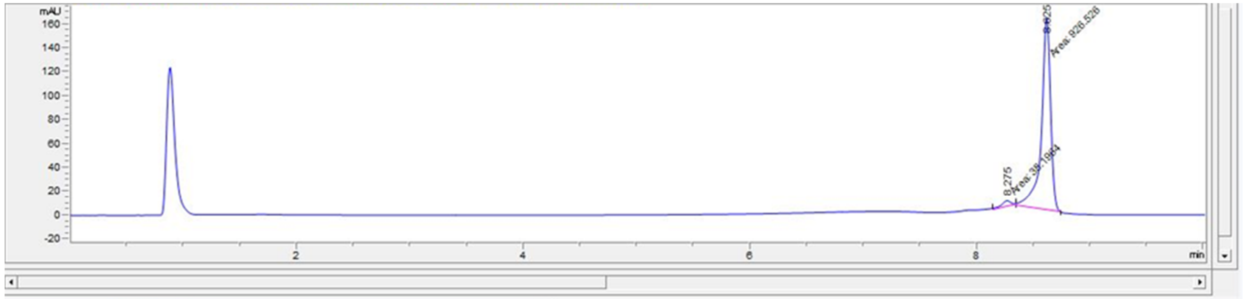


**File Information**

| | |
|---|---|
| LC-File | 1AC-0301.D |
| File Path | D:\data\RD\[LC]\20171030_LC-E638231-1.2.3.4_LC-E63932_LC 20 |
| Date | 30-Oct-17, 17:52:20 |
| Sample | LC-E638231-3 |
| Sample Info | |
| Barcode | |
| Operator | SYSTEM |
| Method | 10%~90%MeOH_COMPOUNDS.M |

| # | Time | Area | Height | Width | Area% | Symmetry |
|---|---|---|---|---|---|---|
| 1 | 8.275 | 38.2 | 5.5 | 0.1157 | 3.959 | 1.533 |
| 2 | 8.625 | 926.5 | 162.1 | 0.0953 | 96.041 | 1.38 |

Conditions for LC-MS/MS analysis

- Agilent Technologies 1200s
- Column: Phenomenex Kinetex 5 μm C18 100A 100 x 2.1 mm
- Flow: 0.3 mL/min
- Detector: PDA detector (315 nm)
- Mobile phase: Water-MeOH gradient

| Time | MeOH |
|---|---|
| 0.0 | 10 |
| 10 | 90 |

**Purity : 96.041%**

**Compound 7**

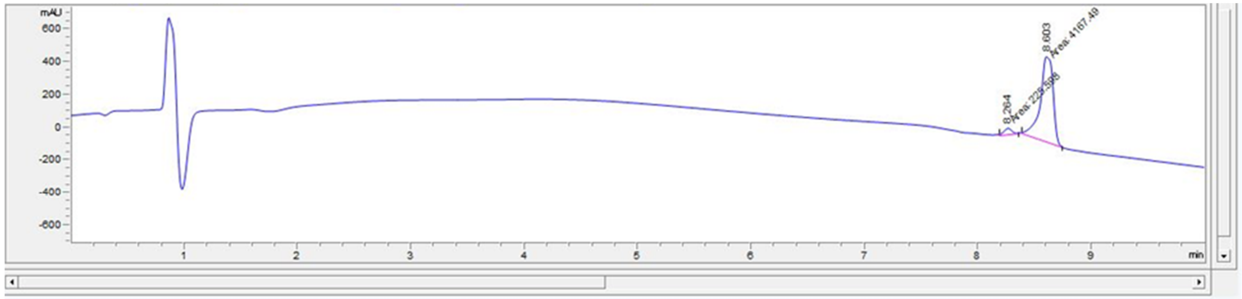


Conditions for LC-MS/MS analysis

- Agilent Technologies 1200s
- Column: Phenomenex Kinetex 5 µm C18 100A 100 x 2.1 mm
- Flow: 0.3 mL/min
- Detector: PDA detector (210 nm)
- Mobile phase: Water-MeOH gradient

**File Information**

| | |
|---|---|
| LC-File | 1AD-0401.D |
| File Path | D:\data\RD\[LC]\20171030_LC-E638231-1.2.3.4_LC-E63932_LC 20 |
| Date | 30-Oct-17, 18:13:21 |
| Sample | LC-E638231-4 |
| Sample Info | |
| Barcode | |
| Operator | SYSTEM |
| Method | 10%~90%MeOH_COMPOUNDS.M |

| # | Time | Area | Height | Width | Area% | Symmetry |
|---|---|---|---|---|---|---|
| 1 | 8.264 | 225.6 | 42.6 | 0.0883 | 5.135 | 0.871 |
| 2 | 8.603 | 4167.5 | 523.6 | 0.1327 | 94.865 | 0.98 |

| Time | MeOH |
|---|---|
| 0.0 | 10 |
| 10 | 90 |

**Purity : 94.865%**

**Compound 8**

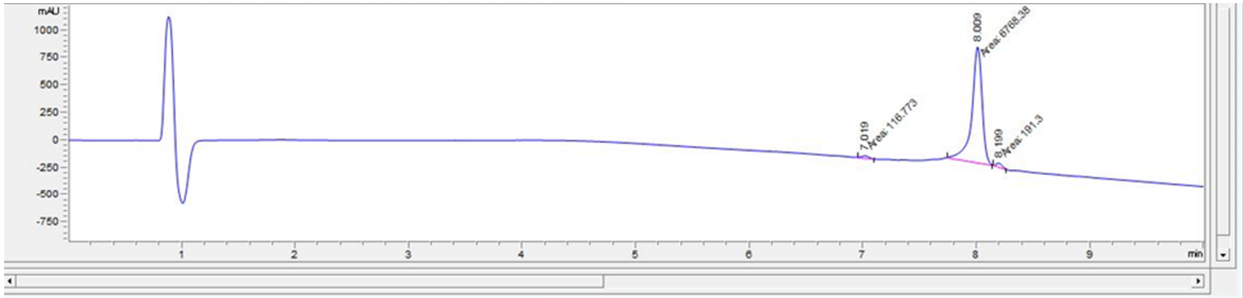


Conditions for LC-MS/MS analysis
- Agilent Technologies 1200s
- Column: Phenomenex Kinetex 5 μm C18 100A 100 x 2.1 mm
- Flow: 0.3 mL/min
- Detector: PDA detector (210 nm)
- Mobile phase: Water-MeOH gradient

**File Information**

| | |
|---|---|
| LC-File | 2CA-0101.D |
| File Path | D:\data\RD\[LC]\20171026_LCE29_LC 2017-10-27 15-14-54 |
| Date | 27-Oct-17, 15:15:57 |
| Sample | LCE29 |
| Sample Info | |
| Barcode | |
| Operator | SYSTEM |
| Method | 10%~90%MeOH_COMPOUNDS.M |

| # | Time | Area | Height | Width | Area% | Symmetry |
|---|---|---|---|---|---|---|
| 1 | 7.019 | 116.8 | 24.8 | 0.0785 | 1.650 | 0.892 |
| 2 | 8.009 | 6768.4 | 1065.8 | 0.1058 | 95.646 | 1.303 |
| 3 | 8.199 | 191.3 | 44.5 | 0.0716 | 2.703 | 0.464 |

| Time | MeOH |
|---|---|
| 0.0 | 10 |
| 10 | 90 |

**Purity : 95.646%**

**Compound 9**
